# Peptides as targeting probes against tumor vasculature for diagnosis and drug delivery

**DOI:** 10.1186/1479-5876-10-S1-S1

**Published:** 2012-09-19

**Authors:** Zhi Jie Li, Chi Hin Cho

**Affiliations:** 1 School of Biomedical Sciences, Faculty of Medicine, The Chinese University of Hong Kong, Hong Kong SAR

## Abstract

Tumor vasculature expresses a distinct set of molecule signatures on the endothelial cell surface different from the resting blood vessels of other organs and tissues in the body. This makes them an attractive target for cancer therapy and molecular imaging. The current technology using the *in vivo* phage display biopanning allows us to quickly isolate and identify peptides potentially homing to various tumor blood vessels. Tumor-homing peptides in conjugation with chemotherapeutic drugs or imaging contrast have been extensively tested in various preclinical and clinical studies. These tumor-homing peptides have valuable potential as targeting probes for tumor molecular imaging and drug delivery. In this review, we summarize the recent advances about the applications of tumor-homing peptides selected by *in vivo* phage display library screening against tumor vasculature. We also introduce the characteristics of the latest discovered tumor-penetrating peptides in their potential clinical applications.

## Background

Up to now, cancer remains one of the leading causes of patients’ deaths worldwide. Successful prevention and treatment of cancer depends on the precise detection at the early stage. Conventional anatomic imaging techniques such as computed tomography (CT) and magnetic resonance imaging (MRI) typically detect tumours when their sizes are bigger than a centimetre in diameter [1,2]. It is evident that more sensitive imaging technologies are needed to be developed to provide early and accurate diagnosis for cancers. Molecular imaging technologies are considered promising methods because they obtain the information through monitoring the key molecular behaviours and host responses related to early events in disease development and progress at the cellular and molecular levels [1,3]. Compared with traditional imaging techniques which are mainly based on anatomical structures of organs, molecular imaging usually utilizes specific molecular probes targeting unique receptors (molecules) of tumor tissues or other diseased tissues to form the localized pictures of image contrast[4]. Thus, it becomes the key point to identify and generate the tumor-specific molecular ligands with high binding affinity.

Likewise, as far as cancer treatment is concerned, targeted drug delivery is attracting intensive attention because it can not only enhance the local drug concentration but also reduce the systemic side effect due to non-specific exposure of anti-cancer drugs to normal tissues. The targeted drug delivery is usually defined as an anti-cancer drug attached by an appropriate tumor-targeting ligand which creates so-called “magic bullet or smart bullet” to produce explosive effects only at the tumor site[5]. Taken together, both of the molecular imaging and targeted drug delivery need tumor-specific ligands to bridge the gap between anti-cancer drug/imaging contrast and tumor tissues. To this end, specific ligands should have the ability to discriminate tumor tissues from normal organs.

Traditionally, antibodies or their fragments are the most common molecular targeting agents for the specific delivery of imaging contrast and anti-cancer drugs to tumor sites. Several monoclonal antibodies have been used in clinics for cancer therapy in the non-conjugated or conjugated manner, such as Trastuzumab (for breast cancer), Bevacizumab (for colorectal cancer), Cetuximab (for colorectal cancer/head and neck cancer) and Ibritumomab tiuxetan (for Non-Hodgkin lymphoma) [5-10]. However, there are two main disadvantages which greatly limit the antibody application, namely the low tumor tissue penetrating ability due to the large size of molecules and nonspecific uptake by the mononuclear phagocyte system (MPS) [6,11]. The advent of peptide library has extended the range of target agents to a great extent and exhibit many unique characteristics when compared with antibody. For instance, peptides display good tissue penetrating ability due to small molecular weight (averagely less than 50 amino acids), low immunogenicity, high affinity to targets, acceptable stability and integrity *in vivo* and easy to manipulate for synthesis and conjugation with other agents [6,11,12]. Phage-displayed peptide library provides us with a possibility to identify and achieve peptide ligands binding to target protein through biopanning the library containing more than billions of peptides. In the past two decades, the phage display technology has undergone a series of important changes and breakthrough developments. Originally, phage peptide library selection was carried out against soluble protein coated in the solid phase. By now, whole cells, tissue samples and live animals have been extensively used as baits to capture feasible binding peptides from a variety of phage libraries [13-15]. These new panning methods are more likely to keep native structure and functional conformation of target proteins than purified protein. Furthermore, they require no previous knowledge of the molecular composition at the site of interest. The peptides so obtained by these methods would possess high affinity and specificity on target sites.

For tumor targeting, ample evidence has indicated that cancer cells and tumor endothelial cells express a distinct set of molecules on their surface that are different from normal cells and blood vessels respectively. This makes cancer cells and tumor vasculature become potential targets for ligand-mediated diagnosis and drug delivery [16,17]. However, what is the better bait for phage peptide screening to identify tumor targeting probes remained to be studied.

### Tumor cells vs tumor vasculature as targets

Cancer cells express a large number of receptors on their surface. Some receptors are overexpressed and mediate important biological functions in tumor growth, migration, invasion and metastasis. Theoretically cancer cell is an excellent target for therapy and imaging. Conventional chemotherapy drugs are mainly designed to target cancer cells. However, it is difficult for these drugs to be absorbed by cancer cells because they can seldom accumulate into tumor mass due to poor blood perfusion, high interstitial pressure and abnormal vasculature inside the tumor mass[18,19]. In fact cancer cells are genetically unstable and often produce multidrug resistance to multiple chemotherapeutic drugs which is also considered as one of major reasons for the failure of cancer therapy [20-22]. Cultured cells which are usually utilized as target for peptide screening might lost tissue-specific characteristics or abnormally express some molecules that do not actually exist in the corresponding cells *in vivo*[23,24]. In contrast with tumor cells, endothelial cells of tumor display several advantages which promote tumor vasculature an attractive target for peptide discovery [5,18,25,26]. Tumor endothelial cells have the good genetic stability so that they rarely produce drug resistance. Tumor blood vessels are also highly accessible to any intravenously administered agents. A great quantity of data based on genomics and proteomics approaches have implicated that endothelial cells (from tumor mass or other organs) express distinct patterns of molecules regulated by their original organ tissues and microenvironment, which are the most important factors for selectivity and a prerequisite for phage peptide selection [27-29]. The unique zip codes of vasculature in different tissues exert vital roles in organ-specific physiological function or disease/tumor development[16].

### *In vivo* phage display

Peptides recognizing specifically the tumor vasculature are promising agents to efficiently deliver drugs and imaging contrast to tumor sites. Tumor angiogenesis, the sprouting and growth of new blood vessels, is indispensible for tumor growth, development and progression [30,31]. Neovascularization of tumors not only provides nutrients and oxygen necessary to tumor growth through blood supply, but also carry cancer cells to adjacent/distant organs to induce metastasis [30,32]. It is likely that destruction of tumor vasculature will halt the tumor blood supply and further restrict tumor progression. *In vivo* phage peptide selection pioneered by Ruoshlahti’s laboratory since 1996[13] has been extensively exploiting the discovery of peptides targeting the vasculature in normal organs or angiogenesis-related diseased tissues. In brief, the procedure of *in vivo* phage library selection is closely similar to the classical *in vitro* screening based on the “binding-washing-eluting-amplifying” process against purified proteins. Only the target is now changed into live animal model. The phage peptide library is intravenously injected into animals and allowed to circulate for 5-15 minutes. It is understood that phage cannot leave the circulation in such a short period of time and the homing peptides displayed on the phage coat protein are able to bind to the target molecules expressed on the endothelial cells in the blood vessels. The subsequent phage enriching screening would allow the target phage to selectively bind to tumor blood vessels [11,27,33]. Using this method numerous peptides targeting the tumor vasculature have been identified. Some of the peptides are also found to recognize cancer cells besides tumor blood vessels owing to the fact that they may share the similar and related receptors in endothelial and cancer cells. Therefore these peptides may serve additional advantages as excellent candidates for drug delivery and cancer treatment [18,34-36].

### Peptides targeting tumor vasculature

It has been shown that peptides obtained from the *in vivo* phage library selection against various tumor vasculatures are capable to efficiently deliver drug/imaging contrast to tumor sites. To this end some peptide conjugates are being tested in clinical studies and achieved promising results. Table 1 gave a summary of tumor vasculature-homing peptides discovered in recent years through *in vivo* phage library screening.

**Table 1:** Peptides targeting tumor blood vessels

Sequence (no. of amino acids, name)	Tumor types tested	Receptors	Applications	References
CDCRGDCFC(9, RGD)	Various tumor types	α_v_β_3_ and α_v_β_5_ integrins	Targeted diagnosis and therapy(TD and TT)	[37,44]
CNGRCVSGCAGRC(13, NGR) CNGRC(5, NGR-2C)	Various tumor types	CD13	TD and TT	[36,37]
CTPSPFSHC(9, TCP-1)	Orthotopic colorectal cancer and gastric cancer	ND*	TD and TT	[19]
IFLLWQR(7, IF7)	Melanoma Colorectal cancer	Anxa1	TT	[60]
CTTHWGFTLC(10)	MDA-MB-435-derived breast carcinomas KS1767-derived Kaposi’s sarcomas	MMP-2 MMP-9	TT	[95]
KDEPQRRSARLSAKPAPPKPEPKPKKAPAKK (31, F3)	HL-60 human leukemia tumor MDA-MB-435 tumors	Nucleolin	ND	[35,65]
CSRPRRSEC(9)	HPV16-induced dysplastic skin			
CGKRK(5) and CDTRL(5)	HPV16-induced skin carcinoma Breast carcinomas	ND	ND	[58]
CKAAKNK(7, KAA) and CKGAKAR(7, KAR)	Pancreatic tumors	ND	ND	[59]
CRGRRST(7,RGR)	Pancreatic tumors Angiogenic islets	PDGF-β	ND	[59]
CRGDK/*R*GPD/*E*C (9, iRGD)	Various tumor types	αv integrins and neuropilin-1	TD and TT	[63,64]
CPRECESIC(9)	EF43-*fgf4*-derived breast tumor MDA-MB-435-derived breast tumor	Aminopeptidase A	TT	[96]
CGNSNPKSC(9, GX1)	Gastric cancer	ND	TD and TT	[97]
SVSVGMKPSPRP (12, SP5-52)	Several cancers	ND	TT	[56]

*Not determined

### Directed drug delivery by targeting-vasculature peptides

Arginine-glycine-aspartic acid (RGD) and asparagine-glycine-arginine (NGR) peptides are the two most famous peptides targeting tumor vasculature discovered earlier by phage display. RGD peptide can home to tumor vasculature selectively expressing α_v_β_3_ and α_v_β_5_ integrins, and NGR peptide (CNGRC) binds to CD13 specifically expressed in tumor vasculature [37]. Although intergrins are also expressed in normal tissue cells and blood vessels, endothelial cells in angiogenic vessels express a distinct set of integrins from its repertoire in quiescent endothelial cells [38]. α_v_β_3_ and α_v_β_5_ integrins are specifically upregulated in tumor endothelial cells undergoing angiogenesis [38,39]. The RGD peptide with two internal disulfide bonds recognizes selectively to α_v_β_3_ and α_v_β_5_ integrins, which promote RGD peptide or its conjugations to accumulate in tumor vasculature [40]. Similarly, CD13 is not exclusively expressed in tumor neovasculature. But NGR peptide specifically targets CD13 molecule expressed in tumor blood vessels rather than other CD13-rich tissues [36,41]. The exact mechanism so far remains unknown, but it is speculated that a unrevealed CD13 isoform is expressed in tumor blood vessels which might be involved in different glycosylation modification or conformational changes[36,42].

The two peptides have become a classic tool to be used to deliver various anti-cancer drugs including but not limited to chemotherapeutic drugs, cytokines, toxins, nucleic acids, radioactive isotopes and nanoparticles [37,43-46]. RGD and NGR peptides coupled with doxorubicin can specifically induce destruction of tumor vasculature in the breast cancer xenograft model in nude mice and further inhibit tumor growth but have no obvious cytotoxic effect on the vasculature of control organs. When conjugated with a pro-apoptotic peptide _D_(KLAKLAK)_2_ which by itself has no activity outside the cells, RGD/NGR peptides could deliver this pro-apoptotic peptide into the tumor vasculature through respective receptors. As a result, the conjugate selectively induced apoptosis of tumor endothelial cells and reduced the tumor growth as well as prolonged survival rate in animals [44]. NGR peptide (CNGRC) was also fused with human tumor necrosis factor alpha (TNF alpha) protein to constitute a new recombinant protein which could greatly enhance the activity of TNF alpha at very lower dose (0.3 μg) in tumor-bearing mice [47]. Further investigation disclosed that subnanogram (0.1 ng) of NGR-TNF alpha could synergistically enhance the tumor toxicity of doxorubicin and melphalan, cisplatin, paclitaxel, and gemcitabine in mouse tumor models, but did not increase the side effects [46]. Phase 1b study of NGR-TNF alpha was tested at low dose in combination with doxorubicin to patients with advanced solid tumors. Results showed that NGR-TNF alpha seldom induced a dose-limited systemic toxicity which previously limited TNF alpha usage for systemic treatment. Patients could well tolerate the side effects when TNF alpha and NGR combined [48]. Phase 1 clinical trial also showed similar conclusions [49]. Similarly, RGD peptide was also proven to be able to significantly enhance anti-tumor activity of TNF alpha at subnanogram level when combined with melphalan in tumor-bearing mice [50]. In addition to TNF alpha, other important cytokines such as truncated tissue factor (tTF), interferon gamma (IFN gamma) and interleukin-12(IL-12) which have great potential as anti-cancer agents, have been fused with NGR/RGD peptides or other peptides targeting tumor blood vessels to enhance their anti-cancer activity [51-55].

In recent years, other novel peptides exhibiting the homing ability to tumor blood vessels were also reported from different laboratories worldwide and tested in various systems [56-60]. Recently, we identified a vasculature-targeting peptide TCP-1 (CTPSPFSHC) using the *in vivo* phage library selection against an orthotopic colorectal cancer model. TCP-1 peptide can specifically recognize the blood vessels of orthotopic colorectal cancer in normal BABL/c mice induced by syngeneic colon cancer cells (colon 26) [19] and also orthotopic gastric cancer in nude mice induced by human gastric cancer cells (unpublished data). We also illustrated that TCP-1 peptide could efficiently deliver the fluorescein and the apoptosis-inducing peptide _D_(KLAKLAK)_2_ to the tumor site for imaging and targeted therapy. TCP-1 peptide appears to be a promising agent in molecular imaging and drug delivery for human gastrointestinal cancers since preliminary data shows that it could also recognize the blood vessels in colorectal cancer samples in humans.

It is envisaged that peptides simultaneously recognizing the tumor vasculature and cancer cells are more effective for cancer therapy than those only targeting tumor blood vessels or cancer cells. Such peptides like NGR and GRD display a dual targeting ability. TCP-1 peptide was also found to be able to bind to some colorectal cancer cells (unpublished data). This kind of peptide conjugate therefore not only can exert targeted anti-cancer function but also provide selective anti-angiogenesis therapy. However, in order to achieve both actions the dose of the new conjugate should be large so as to be able to fully infiltrate into the tumor mass which has a high interstitial pressure and abnormal vasculature structure. The conjugate may only penetrate three to five cell diameters close to blood vessels [18,61]. Recently, the discovery of tumor-penetrating peptides might hold good promise for increasing overall tumor accumulation and penetration abilities of drugs to a great extent. The peptides containing the consensus motif R/KXXR/K (R: arginine, K: lysine, X: any amino acids) [24] can bind to neourophilin-1 (NRP-1) protein expressed on the cell surface. The arginine or lysine in the C-terminal is indispensible for the binding activity. The peptide internally containing the motif can be cleaved by protease to expose the C-terminal so that the binding activity to NRP-1 is switched on (termed C-end rule, CendR). NRP-1 is a cell-surface receptor expressed in various cells and is also found to be overexpressed in tumor tissues [62]. Normally NRP-1 is involved in angiogenesis, regulation of vascular permeability, and development of the nervous system. It is suggested that CendR peptide can increase the vascular permeability, penetrate tissues *in vivo* through binding to NRP-1 of endothelial cells after intravenous injection [24]. The peptide CRGDKGPDC (termed iRGD) obtained through the *in vivo* phage library screening against experimental metastasis mouse model of human prostate cancer contains both RGD and CendR (RGDK) motifs [63]. Further studies showed that i.v. injected iRGD was first recruited to tumor endothelial cells through RGD motif interacting with integrins, and subsequently the CendR motif exposed by the proteolytic cleavage finished the interaction with NRP-1 which drove the peptide to cross the vascular wall and penetrate into the tumor parenchyma. iRGD peptide chemically conjugated with abraxane (a 130 nm albumin-based nanoparticle embedded paclitaxel) could increase more than 4-fold drug accumulation in tumor tissues when compared to conventional RGD conjugates [63]. Moreover, co-administration of the iRGD peptide with various anti-cancer drugs but not chemical conjugation also highly enhanced the drug penetration and accumulation and improved their therapeutic index [64]. Actually, several early-discovered peptides may have the tumor-penetrating activity similar to iRGD because they contain a potential CendR motif, such as F3 (a 31 amino acid peptide homing to cancer cells and tumor endothelial cells) [65], LyP-1(CGNKRTRGC), homing to cancer cells, tumor macrophages and lymphatic vessels [66], CREKA and CSRPRRSEC (two peptides homing to tumor blood vessels) [58,67].

### Directed molecular imaging by targeting-vasculature peptides

The tumor-homing peptides have been investigated in various imaging detection system for tumor diagnosis including MRI, positron emission tomography (PET), single photon emission computed tomography (SPECT) and fluorescence confocal microendoscope when they are coupled with different dyes or imaging agents [68]. RGD and NGR are extensively used to deliver different imaging agents for molecular imaging studies in various tumor types or other angiogenesis diseases. [18F]-labelling RGD conjugate has been developed for PET diagnosis and administered to patients with squamous cell carcinoma of the head and neck (SCCHN) [69]. Results showed that [18F]-labelling RGD can successfully produce specific imaging in α_v_β3-expression SCCHN patients and the conjugate may be useful to assess angiogenesis and response of α_v_β3-targeted therapies for patients with SCCHN [69]. Other phase I trial of PET based on 18F-AH111585/RGD conjugate has been performed in breast cancer patients [70]. The conjugate is retained in the tumor tissues and can detect the breast cancer by PET. Many other agents have also been coupled with RGD peptide for imaging detection such as (64)Cu, (68)Ga [71], near infrared fluorescent [72] and 99mTc [73]. Recently, RGD or NGR peptide coupled with nanoparticles (quantum dots) was tested in mice tumor model for molecular imaging [74-76]. Intravenous injection of NGR peptide-labelled paramagnetic quantum dots (NGR-pQDs) into tumor-bearing mice was used to evaluate the angiogenesis activity of tumors by the MRI system [76]. NGR-pQDs were found to be capable of specifically detecting the tumor region with the highest angiogenic activity [76].

TCP-1 peptide identified by our laboratory was labelled by FITC and administered to mice bearing orthotopic colorectal tumor for *ex vivo* detection under the blue light. The simple conjugate was found to accumulate within the tumor mass with a high specificity, and even it could produce obvious signal in tumor mass as small as 2 millimetres in the *ex vivo* level. If this situation can be reproduced in clinical setting, TCP-1 peptide conjugate combined with PET, MRI or endoscope may improve the diagnosis of patients with colorectal cancer[19].

For the newly discovered peptide iRGD, the iRGD peptide-linked superparamagnetic iron oxide nanoworms can produce hypo-intense vascular signals and low intensity regions in the tumor mass detected by MRI after intravenous injection into tumor-bearing mice[63]. Its effect is obviously better than the conventional RGD peptide for tumor visualization. Histological staining confirms that iRGD nanoworms have the ability to more deeply penetrate into the tumor tissues than the RGD conjugate, suggesting the great potential of iRGD as a diagnostic agent in clinical practice[63].

### Several points worthy of being considered on tumor vasculature-targeting peptides

To date, the mouse tumor model of subcutaneous inoculation is the most frequently applied in cancer-related studies. For the discovery of tumor-homing peptides, a series of peptides have been successfully identified through selecting the phage library *in vivo* against subcutaneous xenograft model in immunodeficient mice [56,77,78]. However, subcutaneous cancer xenograft model cannot actually mimic the complex microenvironment of organs from which cancer cells originate. Thus, the interpretation of experimental results conducted under these conditions sometimes suffers from significant limitations, especially for the identification of vasculature-targeting peptides. It is because microenvironment is actually a vital element which regulates the balance of pro- angiogenic and anti-angiogenic cytokines and determines the angiogenic heterogeneity in tumor mass [79-81]. A growing number of literatures have indicated that various cancer cells interplay with their surrounding microenvironments to influence angiogenesis, cancer cell proliferation, apoptosis, invasion and metastasis [82-85]. A typical example is that human renal cell carcinoma inoculated orthotopically in the kidney of nude mice can produce 10-20 fold higher level of bFGF mRNA than those subcutaneous tumors induced by the same cell line, and orthotopic tumor is highly metastatic and vascularized [86]. Therefore, in our opinion, the orthotopic tumor model is more suitable to select the vasculature-homing peptides to a greater extent. Chances are that more compatible peptides to species from mice to humans can be discovered by this condition. In fact the TCP-1 peptide isolated by our laboratory was found not to target the colorectal tumor induced subcutaneously by the same cell line or other colorectal cancer cells except to recognize the vasculature of orthotopic colorectal cancer (induced by mouse cancer cell in normal BALB/C mice) [19]. Similar findings have also been reported in two other studies by Ruoslahti’s lab [58,59]. They isolated three vasculature-homing peptides against a mouse model of HPV16-induced epidermal carcinogenesis that could distinguish the progressive vascular changes based on this model. But none of them could home to the vasculature of angiogenic dysplasia or tumor in the RIP-Tag transgenic mouse model of pancreatic islet carcinoma whose tumors were located at different site with tumor model used in the course of selection. On the other hand, they variably targeted the vasculature of other tumor types growing in or under the skin [58]. Similarly, other study also found that five peptides homing to neoplastic lesion in the pancreas could not target the subcutaneous islet cell tumor induced by a human cancer cell line or other tumors developed subcutaneously [59]. All these data imply that the microenvironment plays a crucial role in the development and expression of phenotypes in the tumor vasculature.

Tumor microenvironment is often infiltrated by various inflammatory cells such as lymphocytes, mast cells, neutrophils and macrophages which can secrete a variety of cytokines, growth factors and chemokines [82]. These inflammatory mediators are involved in the tumor angiogenesis to a great extent. It is noted that chronic inflammation often accompanies with tissue regeneration and angiogenesis, and even increases the risk of certain cancers. There is no surprise that some tumor vasculature-homing peptides also bind to blood vessels of some inflammatory diseases because of the sharing distribution of certain biomarkers between tumors and inflammatory tissues. For instance, RGD and NGR peptides can also home to blood vessels of collagen-induced arthritis [87], hypoxia-induced retinopathy [88] and ischemic heart [89], all of which are associated with inflammation and angiogenesis. Undoubtedly these findings extend further the scope of application for these peptides. However, under certain circumstances, tumor might coexist with diseases associated with inflammation and angiogenesis such as cerebral ischemia, myocardial infarction and arthritis. Tumor-targeted therapy to destruct the blood vessels might be not suitable in this situation. Therefore, tumor type-specific homing peptides would be more likely to solve the problem. In our study, we found that TCP-1 peptide could not recognize the neovasculature of acute and chronic colitis in mice induced by dextran sulfate sodium (DSS) though chronic inflammatory bowel diseases are directly related to colorectal cancers (unpublished data), suggesting the binding site of TCP-1 peptide in the blood vessels is a marker for tumors but not for inflammatory tissues in the same organ.

The binding sites or receptors of tumor-homing peptides are another important and interesting issue to be studied because the receptor identification would likely produce new biomarkers for tumor diagnosis and therapy. This could lead to a new mechanism for tumor angiogenesis and development. To this end new ligands can be generated to target at this mechanism. However, in contrast with quick discovery of many tumor-homing peptides, identification of receptors is slower and relatively difficult. Only a small number of homing peptides are found together with their receptors through protein pull-down assay or affinity chromatography combined with protein mass spectrometry [24,90-92]. Intrinsic characteristics of membrane protein might be responsible for the low success rate for receptor identification. It is likely that there may be difficult to prepare these binding sites. The low solubility and the destruction of structural conformation of these binding sites may lead to a decrease of ligand affinity during tissue preparation. The other possibility that cannot be excluded is that the receptors might not be proteins, but the motifs of lipids or carbohydrates located on the cell surface.

Finally, nanoparticles applied in cancer diagnosis and therapy may have resulted in the advent of a novel field ‘nanomedicine’. Nanoparticles as drug delivery vectors or imaging probes have been developed and exhibit many superior properties such as better tumor penetrating ability, high capability to carry payloads of therapeutic agents, high quality of imaging information and limited toxicity [93,94]. Nanoparticles combined with tumor-homing peptides can further enhance the targeting ability of nanoparticles and produce more efficient anti-cancer effect and more specific imaging information, which might represent a promising and attractive direction for tumor-targeted diagnosis and therapy.

## Conclusions

To sum up, *in vivo* phage peptide selection provides us with great opportunities to isolate peptides homing to blood vessels of diseased tissues. Since its inception in 1996 creatively developed by Erkki Ruoshlahti[13], numerous vasculature-homing peptides have been identified and tested widely in different models with various interests. On the other hand, these peptide discoveries also prove the concept of vascular zip codes that the endothelial cells in each organ express a distinct set of cell surface molecules. Tumor-homing peptide studies greatly extend the scope of tumor-targeted diagnosis and therapy and produce novel tools as target probes to efficiently and specifically deliver drugs and imaging agents to tumor sites. Identification of tumor-penetrating property of these peptides further extends their potential for tumor-targeted diagnosis and therapy.

## Abbreviations

CT: computed tomography; IFN gamma: interferon gamma; IL-12: interleukin-12; MPS: mononuclear phagocyte system; MRI: magnetic resonance imaging; NGR: Asparagine-glycine-arginine; NGR-pQDs: NGR peptide-labelled paramagnetic quantum dots; NRP-1: neurophilin-1; PET: positron emission tomography; RGD: arginine-glycine-aspartic acid; SCCHN: squamous cell carcinoma of the head and neck; SPECT: single photon emission computed tomography; TNF alpha: tumor necrosis factor alpha; tTF: truncated tissue factor;

## Competing interests

The author(s) declare that they have no competing interests.

## Authors' contributions

ZJL and CHC contributed equally to this manuscript. All authors read and approved the final manuscript.
